# Shorter Telomere Length is Associated with Food Insecurity in Older People: A Cross-Sectional Study

**DOI:** 10.2174/0118746098320942240924074044

**Published:** 2024-10-09

**Authors:** Celi Macedo Polo, Tábatta Renata Pereira de Brito, Wanderson Roberto Silva, Daniela Braga Lima, Daniella Pires Nunes, Fabio Antonio Colombo, Ariene Angelini dos Santos Orlandi, Ligiana Pires Corona

**Affiliations:** 1 Nutrition School, Federal University of Alfenas, Alfenas, Brazil;; 2 Postgraduate Program in Food, Nutrition and Food Engineering, Sao Paulo State University (UNESP), Araraquara, São Paulo, Brazil;; 3 Nursing school, University of Campinas, Campinas, Brazil;; 4 Pharmaceutical Sciences School, Federal University of Alfenas, Alfenas, Brazil;; 5 Nursing Department, Federal University of São Carlos, São Carlos, Brazil;; 6 Department of Applied Sciences, University of Campinas, Campinas, Brazil

**Keywords:** Aged, telomere length, food insecurity, public health, cross-sectional study, nutrition surveys

## Abstract

**Background:**

Telomere length has been investigated as a biomarker of biological aging and is associated with several diseases, lifestyle, and socioeconomic factors.

**Objective:**

This study aimed to verify whether food insecurity is associated with shorter telomere length in older people.

**Methods:**

This is a cross-sectional study carried out in a municipality in the interior of Brazil, with a sample of 440 older people from the community. For telomere length analysis, a blood sample was obtained from each participant, followed by real-time qPCR, and sociodemographic and health information was collected through interviews. Food security/insecurity was measured using the reduced version of the Brazilian Food Insecurity Scale. Descriptive analysis and multiple logistic regression were performed to analyze the factors associated with shorter telomere length, adopting a significance level of 5%.

**Results:**

We found that food insecurity was significantly associated with shorter telomere length, regardless of age group, skin color, tabagism, physical activity, milk and dairy consumption, living arrangement, and basic activities of daily life.

**Conclusion:**

The findings show the importance of ensuring full access to adequate nutrition for the older population, who are physiologically and socially vulnerable.

## 1 INTRODUCTION

With the increasing proportion of older people in the population, research is necessary to better understand the factors associated with aging, so that these individuals have a better quality of life. Leukocyte telomere length (TL) is a measurement that has been used in several studies as a biomarker of biological aging, as there is a tendency for it to progressively reduce throughout life [1]. Telomeres are nucleoprotein structures of non-coding DNA located at the end of the chromosome of eukaryotic cells, which help protect DNA, preventing the loss of genetic material [2]. In addition to maintaining chromosomal integrity, they are essential for cell replication, shortening each event [3]. TL is affected by genes and cumulative exposure to environmental and lifestyle factors. These factors are believed to alter length through their effects on oxidative stress and systemic inflammation [4].

In addition to being associated with the individual's age, TL is also associated with chronic diseases, such as diabetes, gastric cancer, Alzheimer´s disease, and chronic stress [5, 6]. Lifestyles, such as physical activity, diet, and meditation [2, 7, 8], and socioeconomic factors also influence TL [9]. Food insecurity (FI), that is, the lack of access to food both quantitatively and qualitatively, is related to several factors, but mainly to socioeconomic issues [10]. In older people, it is associated with low family income, low educational level, marital status, greater number of depressive symptoms, being a non-white woman, being between 60 and 74 years old, having two or more poor health conditions, being a smoker, among others [11].

Considering the physiological and economic vulnerability of older people, this is a population susceptible to FI, and it is necessary to investigate the impacts of the lack of adequate access to food on the individual's health. Scientific literature has extensively explored the influence of diet on telomeric health [12-14]; however, research on the impact of FI on TL is scarce [15, 16]. Therefore, the objective of this work was to verify whether FI is associated with lower TL in older people in the community.

## 2 MATERIALS AND METHODS

### 2.1 Study Design and Sample Size

This is a quantitative study with a cross-sectional analytical design carried out with older people living in the urban area of Alfenas, a Brazilian municipality with almost 80 thousand inhabitants [17]. To calculate the sample, the G* power statistics was used [18]. The sample size was obtained using the formula *n*=[EDFF*Np(1-p)]/[(d2/Z21-α/2*(N-1)+p*(1-p)], considering estimated proportions of around 50%, 95% confidence interval, a design effect of 1.17 and population of 10,797 older people, which resulted in a sample size of 435 individuals. Fig. (**1**) illustrates the sample definition. The structure of the work followed the guidelines presented in the initiative STROBE (Strengthening the Reporting of Observational Studies in Epidemiology) [19].

### 2.2 Participants

The inclusion criteria adopted were being aged 60 or over and autonomy to provide answers (determined by the interviewer based on the participant's ability to answer their own data - name, date of birth, address, and telephone number). Data from 440 participants were used in the analysis of the present study.

### 2.3 Measures

The information used in the present study that comprised the variables of interest were: gender (male; female), age group (60 to 69 years; 70 to 79 years; 80 years or more), per capita family income in the minimum wage category (> 1; from ½ to 1; < ½), years of education (≤4 years of education, >4 years of education), skin color (white, brown or black), living arrangement (living alone; living with other people), multimorbidity (no/yes), self-perceived health (very good/good; fair; bad/very bad), physical activity, tabagism, alcohol consumption and food consumption (daily intake of milk and dairy products, meat, fish and poultry, daily intake of two or more servings of fruits or vegetables and weekly consumption of two or more servings of beans or eggs - questions obtained from MNA – Mini-exam of Nutritional Assessment) [20]. The presence or absence of signs of anorexia was also verified using the Simplified Nutritional Appetite Questionnaire (SNAQ) [21], self-perceived depressive symptoms using the short version of the Geriatric Depression Scale (GDS) [22, 23], cognitive decline assessed by Cognitive Abilities Screening Instrument – Short Form (CASI-S) [24, 25], basic activities of daily living – BADL – (independence; dependence) assessed by the Katz scale [26] and screening for FI investigated by the abridged version of the Brazilian Food Insecurity Scale (*EBIA*).

The reduced version of *EBIA* is a scale adapted and suggested by Santos *et al.* [27] for FI tracking. This version has five questions that address the individual's concern with the amount of food available at home, as well as their financial condition to access it. The questions do not refer to the presence of children under 18 years of age in the household and are answered using a dichotomous scale (yes/no) considering the previous three months. Participants who answered “yes” to at least one question were evaluated as food insecure.

To analyze leukocyte telomeres, blood samples from each participant were collected in a clinical analysis laboratory and sent to a molecular biology laboratory. For this, the procedure adopted by Cawthon [28] was followed. After DNA extraction with affinity columns, the genetic material was measured in the Genova Nano spectrophotometer (Jenway), with the concentration corrected to 10 ng/μl in the samples, which were frozen at -20°C until the time of PCR (Polymerase Chain Reaction). To identify telomeres, telg 5’ ACA CTA AGG TTT GGG TTT GGG TTT GGG TTT GGG TTA GTGT3’ and telc 5’ TGT TAG GTA TCC CTA TCC CTA TCC CTA TCC CTA TCC CTA ACA3’ markers were used. For the control gene (human betaglobulin), the markers hbgu 5’CGG CGG CGG GCG GCG CGG GCT GGG CGG ctt cat cca cgt tca cct tg3’ and hbgd 5’GCC CGG CCC GCC GCG CCC GTC CCG CCG gag gag aag tct gcc gtt3’ were used.

The average relative telomere length was calculated using a mathematical model [28] and categorized into quartiles [29]. Older people with a TL value in the lowest 25% were classified as having “shorter telomere length”.

### 2.4 Procedures and Ethical Aspects

Data were collected in different regions of the municipality of Alfenas between July and December 2019 through interviews carried out at each participant's home. The interviews were conducted by trained researchers, and each lasted an average of one hour. For the selection of participants, the same sample complementation method developed during the SABE study (Health, Well-being, and Aging) was used, and a population-based survey was carried out in the city of São Paulo [30]. Therefore, the interviewers were distributed in different regions of the municipality according to the proximity to the region where they live and, after locating a household with a resident aged 60 or over, they proceeded to locate nearby houses or, at most, within the boundaries of the neighborhood to which the initial address belonged.

In the interview, each participant provided responses to the set of items/questions that were previously described in the Measures section. Before the interview, the researcher explained the objectives and procedures of the research to the individual who, upon voluntarily agreement to participate, signed the Informed Consent Form. The research was approved by the Research Ethics Committee of the Federal University of Alfenas under the approval statement Nº 2.668.936/2018 and CAAE 85218518.0.0000.5142.

### 2.5 Data Analysis

Descriptive analysis was used to characterize the study sample. The chi-square test (χ^2^) was used to evaluate the difference in proportions between TL and individual characteristics of the participants (gender, age group, minimum wage per capita, skin color, years of education, living arrangement, self-perceived depressive symptoms, signs of cognitive decline, BADL, self-perceived health, physical activity, tabagism, anorexia, FI and food consumption). Logistic regression models were used to estimate crude and adjusted odds ratios (OR) and respective 95% confidence intervals (CI). Sociodemographic and health covariates that presented *p<*0.20 in the unadjusted analysis were incorporated into the adjusted analysis [31].

The final model was composed only of statistically significant associations (*p<*0.05). To evaluate the quality of the final model, the area under the ROC curve (AUROC) was used. All analyses were performed using Stata^®^ software version 17.0.

## 3 RESULTS

Among the participants, 70.8% reported being female, and the majority reported being aged between 60 and 69 years old (45.3%), having white skin color (60.3%), minimum wage per capita less than 1 minimum wage (60.8%) and self-perceived health very good/good (62.5%).

Towards the profile of older people experiencing FI, it was found that the variables, including skin color, years of education, minimum wage per capita, symptoms of depression and cognitive decline, physical activity, alcohol consumption, anorexia, and daily consumption of meat, fish, and poultry showed different proportions (*p<*0.05) when compared to presence/absence of FI (Table **1**).

Regarding the nutrition of the individuals, 22.1% presented anorexia, and the majority presented qualitative adequacy regarding food consumption. It was observed that 13.6% of participants screened positive for food insecurity. The variables screening for food insecurity (*p=*0.014), anorexia (*p=*0.031), and week consumption of two or more portions of beans or eggs (*p=*0.049) showed different proportions when compared to telomere length (Table **2**).

In univariate analyses (Table **3**), the characteristics of skin color (white *vs.* black: *p=*0.057), minimum wage per capita (> 1 *vs.* < ½: *p=*0.180), living arrangement (living alone *vs.* living with other people: *p=*0.08), screening for food insecurity (negative screening *vs.* positive screening: *p=*0.015), self-perceived symptoms of cognitive decline (absent *vs.* present: *p=*0.152), physical activity (no *vs.* yes: *p=*0.114), tabagism (no *vs.* yes: *p=*0.086), anorexia (no *vs.* yes: *p=*0.032), BADL (independently *vs.* dependently: *p=*0.165) and daily consumption of milk and dairy (no *vs.* yes: *p=*0.068), weekly consumption of two or more servings of beans or eggs (yes *vs.* no: *p=*0.053) were considered eligible for multiple analysis, since these relationships showed *p<*0.20.

In the multiple models, after the inclusion of covariates, FI was associated with TL regardless of age group, skin color, consumption of milk and dairy products, living arrangement, BADL, physical activity, and tabagism (Table **4**). In Fig. (**2**), AUROC represents the quality of the final model, indicating that the significant characteristics were able to explain 65% of the shorter TL.

## 4 DISCUSSION

In our study, we observed that positive screening for FI in the older population was significantly associated with shorter telomere length, regardless of age group, skin color, physical activity, tabagism, milk and dairy consumption, living arrangement, and BADL.

It is well established that diet is related to TL, affecting it in both adults and older people [7, 13, 14]. Considering that FI consists of a lack of access to food quantitatively and qualitatively, a possible explanation for the relationship between FI and shorter TL may be related to the fact that the dietary consumption of different food groups, such as meat, fruits, vegetables, and dairy products in older adults is inadequate [32-33]. In the research on how much the Brazilian family spends on food, it was found that in a food security situation, expenses for fruits and vegetables, meat, offal, and fish and dairy products are higher than when in FI. On the other hand, families in FI spent more on rice and beans [34]. In other words, the lower the access to adequate food, the lower the consumption of foods associated with TL conservation, as described in the literature [7, 13, 35-37]. In our study, we found that individuals with FI consume meat, fish, and poultry less frequently (*p<*0.001). There was also a lower consumption of fruits, vegetables, dairy products, legumes, and eggs in older people in FI compared to those in food security. However, there was no statistically significant difference.

From this information, we can state that the consumption of important food groups (sources of protein, vitamins, minerals, and bioactive compounds) by families in FI is below ideal according to the Dietary Reference Intakes [38], increasing the risk of having a shorter TL [37].

A systematic review of eight cross-sectional studies with older people [39] showed that FI was associated with low intake of vitamins, such as vitamins A, D, E, B2, and B12, and minerals iron, zinc, calcium, and magnesium, in addition to insufficient consumption of energy, macro-and micronutrients.

There are nutrients, such as vitamins C, D, E, folate, and β-carotene, and the minerals zinc and magnesium, which have demonstrated positive effects in protecting telomeres against oxidative stress and inflammation, being positively associated with TL [37].

Considering the information presented, we expect that individuals with FI, with inadequate dietary intake of macro-and micronutrients, will have less protection against cellular oxidative stress.

In a review carried out by Vidaček *et al*. [40] on the relationship between nutrition and telomeres and aging, they found that inadequate nutrition, as well as other unfavorable lifestyle factors, influence the increase in damage to the genome and accelerate telomeric shortening through the formation of free radicals and stress oxidative. Reactive oxygen species (ROS) exert a strong influence on replicative senescence and aging, mainly by causing the accumulation of nicks in the guanine-rich (G) strand, resulting in accelerated telomeric shortening.

It has been observed that healthy eating patterns (high intake of fruits, vegetables, whole grains, dairy products, and vegetable proteins and low intake of red and processed meat, sodium, and added sugars) are related to a higher TL of leukocytes, especially in women [13]. A review carried out by Balan *et al*. presented studies verifying the relationship between diet and TL in adults and older people, verifying that TL is positively associated with the regular consumption of healthy foods such as whole grains, fish, nuts, legumes, seaweed, vegetables, fruits, natural juices, dairy products, coffee and adherence to the Mediterranean diet, which has antioxidant potential [7, 14].

More specifically, on the relationship between telomere shortening and food security, a cross-sectional study carried out in adults and older people participating in the National Health and Nutrition Examination Survey (NHANES) from 1999 to 2002 found a relationship between FS condition and longer leukocyte TL in individuals aged between 25 and 44.9 years. TL of individuals aged between 18 and 24.9 years and over 45 were not associated with FI [16]; that is, FI was not associated with a shorter TL in older people. Seeking to explain the association observed, the authors raise hypotheses related to the low consumption of food sources of antioxidants and vitamins associated with anti-inflammatory and immunomodulatory activities. In a study carried out with data from the same survey (NHANES 1999-2002) but with data from older people (60 to 85 years old), Lima *et al*. [15] found that FI was associated with shorter telomeres only when the sample was stratified according to the level of social support, finding an association between FI and shorter TL in older people with a high level of social support. Unlike previous studies, our study found an association between FI and lower TL, regardless of covariates, providing unprecedented data on this relationship in older people.

In addition to the lower intake of antioxidants from a healthy diet, adequate in quantity and quality, TL is affected by stressors, such as lack of access to food, anxiety, and depression, resulting in a cascade of stress hormones [16]. There is evidence that having difficulty accessing food can negatively affect emotions [41, 42], as well as when mental health is impaired, and this can alter individuals' dietary patterns [43, 44]. Stress from discrimination and poverty-related factors has been associated with adverse effects on TL, with some evidence that stress from poverty can induce telomere attrition [45].

FI can affect individuals from all population groups; however, there is a prominent concern for vulnerable individuals, such as older people [46]. This population already has a lower TL due to aging itself; in a situation of FI, the risk of further reducing TL increases due to factors such as macro-and micronutrient deficits and chronic stress associated with poverty and low income. Therefore, our results on the association of FI and lower TL are important for public managers and health professionals to consider access to food in public policy and clinical conduct, respectively.

This study has limitations. As this is a cross-sectional study, it is not possible to infer a cause-and-effect relationship between the variables studied, with the associations found as warnings of susceptibility and the arbitrary categorization of telomere length, as there are no parameters established in the literature. As this is a larger study, whose main objective was not to thoroughly research the role of diet in TL, one of the limitations was the instruments used to collect information about the diet of older people: food consumption based on questions of MNA and the use of reduced *EBIA*, which does not measure FI in different degrees nor is it a validated instrument for use in older people. However, this instrument is suitable for FI tracking and practical for field research.

To overcome these limitations, we suggest carrying out longitudinal studies to analyze both the FI situation and TL in older people over time. Even with limitations, the findings of the present study show us that the lack of adequate access to food affects health as a whole, as well as accelerates biological aging as demonstrated by the shorter TL.

## CONCLUSION

Food insecurity in older people was associated with shorter telomere length regardless of age group, skin color, tabagism, physical activity, milk and dairy consumption, living arrangements, and BADL. TL is affected by several factors, and FI may contribute to its reduction in at least two ways: through inadequate access to nutrients, qualitatively and quantitatively, and by causing stress to the individual in this situation. The older population, which, due to its age, already has a lower TL than younger adults, in conditions of FI, will present even greater biological aging. Hence, the findings show the importance of ensuring full access to adequate nutrition for the older population, which is physiologically and socially vulnerable.

## AUTHORS’ CONTRIBUTIONS

The authors confirm their contribution to the paper as follows: The study concept or design was contributed by L.C., A.O., D.N., and T. B.

Data Analysis or interpretation was performed by F.C. and W.S. Data collection was contributed by D.L., and the paper was written by C.P.

## Figures and Tables

**Fig. (1):**
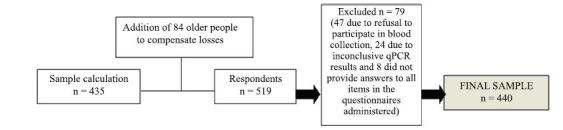
Flowchart of sample definition.

**Fig. (2):**
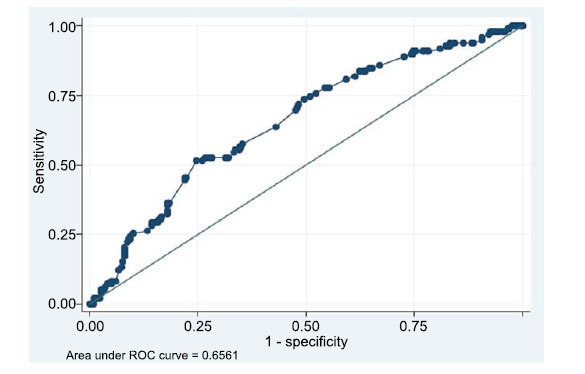
Area under ROC (Receiver Operating Characteristic) curve representing the fit of the final model of logistic regression between individual characteristics of older people and shorter telomere length. The value 0.6561 means that the final model explains 65% of the TL shortening.

**Table 1:** Descriptive analysis and assessment of the difference in proportions between individual characteristics of older people and food insecurity.

**Variables**	***n* (%)**	**Food Insecurity**		***p***
		**No**	**Yes**	
		**380 (86.4%)**	**60 (13.6%)**	
Gender				
Male	127 (28.9)	116 (30.5)	11 (18.3)	0.053
Female	313 (71.1)	264 (69.5)	49 (81.7)	
Age group (years)				
60 – 69	196 (44.5)	171 (45.0)	25 (41.7)	0.870
70 – 79	171 (38.9)	147 (38.7)	24 (40.0)	
80 and older	73 (16.6)	62 (16.3)	11 (18.3)	
Skin color/ethnicity				
White	264 (60.55)	239 (63.4)	25 (42.4)	0.007*
Brown	152 (34.9)	123 (32.6)	29 (49.1)	
Black	20 (4.6)	15 (4.0)	5 (8.5)	
Years of education				
>4	138 (33.6)	132 (37.2)	6 (10.7)	<0.001*
≤4	273 (66.4)	223 (62.8)	50 (89.3)	
Minimum wage per capita#				
> 1	160 (39.1)	152 (43.8)	8 (14.8)	<0.001*
½ - 1	180 (44.0)	153 (43.1)	27 (50.0)	
< ½	69 (16.9)	50 (14.1)	19 (35.2)	
Living arrangement				
Living with other people	354 (81.4)	304 (19.4)	50 (13.8)	0.310
Living alone	81 (18.6)	73 (80.6)	8 (86.2)	
BADL				
Independence	375 (87.2)	325 (87.6)	50 (84.7)	0.241
Dependence	55 (12.8)	46 (12.4)	9 (15.3)	
Self-perceived depressive symptoms				
Absent	287 (65.2)	261 (68.7)	26 (43.3)	<0.001*
Present	153 (34.8)	119 (31.3)	34 (56.7)	
Self-perceived symptoms of cognitive decline				
Absent	310 (70.4)	276 (72.6)	34 (56.7)	0.012*
Present	130 (29.6)	104 (27.4)	26 (43.3)	
Multimorbidity				
No	131 (30.18)	114 (30.32)	17 (29.31)	0.876
Yes	303 (69.82)	262 (69.68)	41 (70.69)	
Self-perceived health				
Very good/Good	274 (62.4)	243 (64.1)	31 (51.7)	0.066
Fair	143 (32.6)	120 (31.7)	23 (38.3)	
Bad/Very bad	22 (5.0)	16 (4.2)	6 (10.0)	
Physical activity				
No	289 (68.97)	241 (66.94)	48 (81.36)	0.027*
Yes	130 (31.03)	119 (33.06)	11 (18.64)	
Tabagism				
No	380 (86.76)	327 (86.51)	53 (88.33)	0.698
Yes	58 (13.24)	51 (13.49)	7 (11.67)	
Alcohol consumption				
No	304 (71.03)	253 (68.75)	51 (85.00)	0.010*
Yes	124 (28.97)	115 (31.25)	9 (15.00)	
Anorexia				
No	342 (77.7)	309 (81.3)	33 (55.0)	<0.001*
Yes	98 (22.3)	71 (18.7)	27 (45.0)	
Daily intake of milk and dairy products				
Yes	305 (69.3)	269 (70.8)	33 (60.0)	0.092
No	135 (30.7)	111 (29.2)	24 (40.0)	
Daily intake of meat, fish and poultry				
Yes	327 (74.3)	298 (78.4)	29 (48.3)	<0.001*
No	113 (25.7)	82 (21.6)	31 (51.7)	
Daily intake of 2 or more servings of fruits or vegetables				
Yes	334 (76.3)	292 (77.3)	42 (70.0)	0.220
No	104 (23.7)	86 (22.7)	18 (30.0)	
Weekly consumption of 2 or more servings of beans or eggs				
Yes	404 (92.4)	351 (93.1)	53 (88.3)	0.194
No	33 (7.6)	26 (6.9)	7 (11.7)	

**Note: ** BADL: basic activities of daily living. *p<0.05 by chi-square test. #In 2019 one (01) minimum wage was equal to approximately US$250.00.

**Table 2:** Descriptive analysis and assessment of the difference in proportions between individual characteristics of older people and shorter telomere length.

**Variables**	**Total *n*(%)**	**Shorter TL**		***p***
		**No**	**Yes**	
		**336 (75.0%)**	**112 (25.0%)**	
Gender				
Male	131 (29.2)	101 (30.1)	30 (26.8)	0.509
Female	317 (70.8)	235 (69.9)	82 (73.2)	
Age group (years)				
60 – 69	203 (45.3)	153 (45.5)	50 (44.6)	0.527
70 – 79	172 (38.4)	125 (37.2)	47 (42.0)	
80 and older	73 (16.3)	58 (17.3)	15 (13.4)	
Skin color/ethnicity				
White	264 (60.3)	198 (60.5)	66 (59.5)	0.110
Brown	154 (35.1)	118 (36.1)	36 (32.4)	
Black	20 (4.6)	11 (3.4)	9 (8.1)	
Years of education				
>4	140 (33.9)	105 (33.4)	35 (35.3)	0.720
≤4	273 (66.1)	209 (66.6)	64 (66.7)	
Minimum wage per capita				
> 1	161 (39.2)	126 (41.0)	35 (33.6)	0.354
½ - 1	180 (43.8)	132 (43.0)	48 (46.2)	
< ½	70 (17.0)	49 (16.0)	21 (20.2)	
Living arrangement				
Living with other people	356 (81.5)	261 (79.6)	95 (87.2)	0.078
Living alone	81 (18.5)	67 (20.4)	14 (12.8)	
Food insecurity				
Negative screening	380 (86.4)	291 (88.7)	89 (79.5)	0.014*
Positive screening	60 (13.6)	37 (11.3)	23 (20.5)	
BADL				
Independence	376 (87.0)	276 (85.7)	100 (90.9)	0.161
Dependence	56 (13.0)	46 (14.3)	10 (9.1)	
Self-perceived depressive symptoms				
Absent	289 (65.2)	219 (66.2)	70 (62.5)	0.482
Present	154 (34.8)	112 (33.8)	42 (37.5)	
Self-perceived symptoms of cognitive decline				
Absent	311 (70.5)	238 (72.3)	73 (65.2)	0.151
Present	130 (29.5)	91 (27.7)	39 (34.8)	
Multimorbidity				
No	131 (30.2)	100 (30.9)	31 (28.2)	0.596
Yes	303 (69.8)	224 (69.1)	79 (71.8)	
Self-perceived health				
Very good/Good	275 (62.5)	204 (62.0)	71 (64.0)	0.920
Fair	143 (32.5)	108 (32.8)	35 (31.5)	
Bad/Very bad	22 (5.0)	17 (5.2)	5 (4.5)	
Physical activity				
No	290 (69.0)	211 (67.0)	79 (75.2)	0.113
Yes	130 (31.0)	104 (33.0)	26 (24.8)	
Tabagism				
No	381 (86.8)	290 (88.4)	91 (82.0)	0.084
Yes	58 (13.2)	38 (11.6)	20 (18.0)	
Alcohol consumption				
No	305 (71.1)	223 (69.9)	82 (74.5)	0.355
Yes	124 (28.9)	96 (30.1)	28 (25.5)	
Anorexia				
No	348 (77.9)	269 (80.3)	79 (70.5)	0.031*
Yes	99 (22.1)	66 (19.7)	33 (29.5)	
Daily intake of milk and dairy products				
Yes	306 (69.4)	236 (71.7)	70 (62.5)	0.067
No	135 (30.6)	93 (28.3)	42 (37.5)	
Daily intake of meat, fish and poultry				
Yes	328 (74.4)	247 (75.1)	81 (72.3)	0.564
No	113 (25.6)	82 (24.9)	31 (27.7)	
Daily intake of 2 or more servings of fruits or vegetables				
Yes	335 (76.3)	255 (77.7)	80 (72.1)	0.224
No	104 (23.7)	73 (22.3)	31 (27.9)	
Weekly consumption of 2 or more servings of beans or eggs				
Yes	405 (92.5)	308 (93.9)	97 (88.2)	0.049*
No	33 (7.5)	20 (6.1)	13 (11.8)	

**Note: ** TL: telomere length; BADL: basic activities of daily living. *p<0.05 by chi-square test. #In 2019 one (01) minimum wage was equal to approximately US$250.00.

**Table 3:** Univariate analyses to measure the crude association (odds ratio – OR) and 95% confidence interval (CI) between individual characteristics of older people and shorter telomere length.

**Variables**	***OR***	***p***	***IC95%***
Gender			
Male	1.00		
Female	1.17	0.510	0.72-1.89
Age group (years)			
60 – 69	1.00		
70 – 79	1.15	0.553	0.72-1.82
80 and older	0.79	0.481	0.41-1.51
Skin color/ethnicity			
White	1.00		
Brown	0.91	0.709	0.57-1.45
Black	2.45	0.057	0.97-6.18
Years of education			
>4	1.00		
≤4	0.91	0.726	0.57-1.47
Minimum wage per capita			
> 1	1.00		
½ - 1	1.31	0.290	0.79-2.15
< ½	1.54	0.180	0.81-2.90
Living arrangement			
Living with other people	1.00		
Living alone	0.57	0.080	0.30-1.07
Food insecurity			
Negative screening	1.00		
Positive screening	2.03	0.015*	1.14-3.60
BADL			
Independence	1.00		
Dependence	0.60	0.165	0.29-1.23
Self-perceived depressive symptoms			
Absent	1.00		
Present	1.17	0.482	0.75-1.83
Self-perceived symptoms of cognitive decline			
Absent	1.00		
Present	1.39	0.152	0.88-2.20
Multimorbidity			
No	1.00		
Yes	1.06	0.597	0.84-1.35
Self-perceived health			
Very good/Good	1.00		
Fair	0.93	0.765	0.58-1.48
Bad/Very bad	0.84	0.749	0.30-2.37
Physical activity			
No	1.00		
Yes	0.66	0.114	0.40-1.10
Tabagism			
No	1.00		
Yes	1.67	0.086	0.92-3.02
Alcohol consumption			
No	1.00		
Yes	0.79	0.355	0.48-1.29
Anorexia			
No	1.00		
Yes	1.70	0.032*	1.04-2.77
Daily intake of milk and dairy products			
Yes	1.00		
No	1.52	0.068	0.97-2.39
Daily intake of meat, fish and poultry			
Yes	1.00		
No	1.15	0.564	0.71-1.87
Daily intake of 2 or more servings of fruits or vegetables			
Yes	1.00		
No	1.35	0.225	0.83-2.20
Weekly consumption of 2 or more servings of beans or eggs			
Yes	1.00		
No	2.06	0.053	0.99-4.30

**Note: ** BADL: basic activities of daily living. *p<0.05 by multiple logistic regression. #In 2019 one (01) minimum wage was equal to approximately US$250.00.

**Table 4:** Multiple analyses represent the final model with adjusted association measures (odds ratio – OR) and 95% confidence interval (CI) between individual characteristics of older people and shorter telomere length.

**Variables**	**OR**	***p***	***IC95%***
Food insecurity			
Negative screening	1.00		
Positive screening	1.91	0.042*	1.02-3.57
Age group (years)			
60 – 69	1.00		
70 – 79	1.34	0.271	0.79-2.25
80 and older	0.75	0.447	0.36-1.56
Skin color/ethnicity			
White	1.00		
Brown	0.83	0.493	0.49-1.39
Black	2.00	0.176	0.73-5.48
Living arrangement			
Living with other people	1.00		
Living alone	0.54	0.084	0.27-1.08
Tabagism			
No	1.00		
Yes	1.65	0.157	0.82-3.30
Physical activity			
No	1.00		
Yes	0.74	0.280	0.43-1.27
BADL			
Independence	1.00		
Dependence	0.69	0.350	0.32-1.49
Daily intake of milk and dairy products			
Yes	1.00		
No	1.27	0.346	0.77-2.09

**Note: ** BADL: basic activities of daily life. *p<0.05 by multiple logistic regression followed by stepwise forward.

## Data Availability

The data confirming the conclusions of the article were not deposited in the database. Data can be provided upon request *via* email to the corresponding author: tabatta.brito@unifal-mg.edu.br.

## LIST OF ABBREVIATIONS

AUROC: Area Under Receiver Operating Characteristic
BADL: Basic Activities of Daily Living
CASI-S: Cognitive Abilities Screening Instrument-Short form
CI: Confidence Interval
DNA: Deoxyribonucleic Acid
EBIA: *Escala Brasileira de Insegurança Alimentar* (Brazilian Food Insecurity Scale)
FI: Food insecurity
GDS: Geriatric Depression Scale
MNA: Mini-exam of Nutritional Assessment
NHANES: National Health and Nutrition Examination Survey
OR: Odds ratio
qPCR: Quantitative Polymerase Chain Reaction
SABE: *Saúde, Bem-estar e Envelhecimento* (Health, Well-being and Aging)
SNAQ: Simplified Nutritional Appetite Questionnaire
STROBE: Strengthening the Reporting of Observational Studies in Epidemiology
TL: Telomere Length

## STANDARDS OF REPORTING

STROBE guidelines were followed.
